# Looking for Options to Sustainably Fixate Nitrogen. Are Molecular Metal Oxides Catalysts a Viable Avenue?

**DOI:** 10.3389/fchem.2021.742565

**Published:** 2021-09-14

**Authors:** Rebeca González-Cabaleiro, Jake A. Thompson, Laia Vilà-Nadal

**Affiliations:** ^1^ Department of Biotechnology, Delft University of Technology, Delft, Netherlands; ^2^ School of Chemistry, University of Glasgow, Glasgow, United Kingdom

**Keywords:** nitrogen fixation, polyoxomatalate, nitrogenase, compuatational chemistry, metabolic modelling, Haber Bosch, catalyst—N

## Abstract

Fast and reliable industrial production of ammonia (NH_3_) is fundamentally sustaining modern society. Since the early 20^th^ Century, NH_3_ has been synthesized *via* the Haber–Bosch process, running at conditions of around 350–500°C and 100–200 times atmospheric pressure (15–20 MPa). Industrial ammonia production is currently the most energy-demanding chemical process worldwide and contributes up to 3% to the global carbon dioxide emissions. Therefore, the development of more energy-efficient pathways for ammonia production is an attractive proposition. Over the past 20 years, scientists have imagined the possibility of developing a milder synthesis of ammonia by mimicking the nitrogenase enzyme, which fixes nitrogen from the air at ambient temperatures and pressures to feed leguminous plants. To do this, we propose the use of highly reconfigurable molecular metal oxides or polyoxometalates (POMs). Our proposal is an informed design of the polyoxometalate after exploring the catabolic pathways that cyanobacteria use to fix N_2_ in nature, which are a different route than the one followed by the Haber–Bosch process. Meanwhile, the industrial process is a “brute force” system towards breaking the triple bond N-N, needing high pressure and high temperature to increase the rate of reaction, nature first links the protons to the N_2_ to later easier breaking of the triple bond at environmental temperature and pressure. Computational chemistry data on the stability of different polyoxometalates will guide us to decide the best design for a catalyst. Testing different functionalized molecular metal oxides as ammonia catalysts laboratory conditions will allow for a sustainable reactor design of small-scale production.

## Introduction

Multicellular organisms are unable to metabolize atmospheric N_2_ because of its high bond enthalpy and zero dipole moment. Instead, they source nitrogen from fixed resources such as nitrate and ammonia (Sadeghi et al., 2015). The process known as biological nitrogen fixation in which N_2_ is converted into assimilable forms is carried out by a specialized group of microorganisms that possess nitrogenases which are enzymes able to reduce atmospheric nitrogen into ammonia (NH_3_). At the start of the last century the only solid natural forms of nitrogen to enrich the soil were Peruvian guano and Chilean nitrate but in 1913, the Haber–Bosch process changed the course of the 20^th^ Century allowing mass production of ammonia. In fact, ammonia production is the base of agriculture supporting between a third and a half of human food intake. Despite technical improvements for industrial NH_3_ production, it still requires both high temperature (350–500°C) and high pressure (15–20 MPa) consuming more than 1% of world-wide energy production and being one of the main world-wide producers of carbon dioxide and nitrous oxide emissions, both tagged as green-house gases (Foster et al., 2018). We can reduce travelling to mitigate climate change, but definitely, we cannot stop eating (Erisman et al., 2008), and massive industrial ammonia production of NH_3_ is fundamental in sustaining the human population (50% of the nitrogen found in human tissues originates from the Haber–Bosch process). However, the abuse of ammonia fertilizers, of which only about 50% are efficiently absorbed in soils, has led to an accumulation of nitrogen in natural waterbodies with negative consequences (such as limitation of natural diversity and proliferation of toxic algae) (Fields, 2004). Therefore, sustainable nitrogen fixation has remained as a critical area of research at the frontiers of inorganic, organometallic, coordination chemistry, and biochemistry for decades. Finding efficient alternatives to the Haber–Bosch process is a challenge because of the extraordinarily complicated characteristics of the reaction. In fact, ammonia synthesis is currently the most well-characterized heterogeneous catalytic reaction.

The overall reaction of ammonia synthesis from N_2_ is accessible thermodynamically at standard conditions (ΔG° = −16.4 kJ mol^−1^) (Lide, 2005), which indicates that this reaction could occur without external energy input at low temperatures. However, it does not take place spontaneously (Jia and Quadrelli, 2014). Kinetics, and endergonic production of intermediates, dictate operation at ca 350–500°C and elevated pressures are needed to achieve acceptable process yields at an industrial level (Hargreaves et al., 2020). The detailed thermodynamic analysis presented in (Jia and Quadrelli, 2014) also shows that although the overall reaction of fixing N_2_ is exergonic, the kinetic routes that lead to them demand high amounts of energy. Indeed, diazene and hydrazine are intermediates of the overall reaction with very high enthalpies of formation (Van Der Ham et al., 2014).

Given its global impact, the fundamentals of the Haber–Bosch process have hardly changed at all over the past 100 years. It still relies on an iron catalyst with potassium oxide and alumina acting as electronic and structural promoters, respectively (Galloway et al., 2013). In the early 1900s, Alwin Mittasch conducted a large-scale screening experiment to find a substitute for Haber’s osmium- and uranium-based catalysts (Hargreaves, 2014). Approximately 3,000 catalyst compositions were evaluated in over 20,000 small-scale tests. He developed a Fe-based catalyst, which is still used today, but in the 1970s ruthenium (Ru) was acknowledged as the best elemental metal catalyst for industrial ammonia production.

In recent years, there has been a large amount of research on reducing the temperature and pressure of the Haber-Bosch process using a variety of advanced catalysts such as promoted-iron, supported-ruthenium, and metal nitrides (Humphreys et al., 2021). Today we know that Ru has much higher activity than Fe, at least near thermodynamic equilibrium. However, due to the higher cost of Ru and its shorter catalytic lifetime, promoted Ru catalysts have only recently begun to challenge iron-based catalysts (Ross, 2019). Also, it has been long accepted that d-block metals can bind the abundant dinitrogen molecule, however, only a few are able to catalyze the conversion of dinitrogen to ammonia. Indeed, the main impediment to N_2_ fixation is primarily of kinetic nature (Jia and Quadrelli, 2014). After carefully analyzing existing thermodynamic experimental data, Borden provided an insightful explanation to the energetics of bonding H_2_ to N_2_ (Borden, 2017). The study showed how the difficulty associated to N_2_ fixation, is only partly due to the strength of one of the three N−N π bond that is broken in this reaction. In fact, the relative weakness of the intermediate sp^2^ N−H σ bonds in E-HN = NH obtained in this reaction plays a slightly larger role which allows us to conclude that reactivity of the intermediates rely on a delicate balance between the bonds that are formed and broken towards the yielding of the final product (Nicolaides and Borden, 1991).

Under the current global scenario of environmental emergency, it is urgent to find sustainable solutions to fulfil the ammonia demands of the human population. Novel design of catalysts is required to efficiently produce NH_3_ at low temperatures and with less energy requirements. Ideally, these catalyzers could drive N_2_ fixation at small scale, tailoring the operation for specific demands and contributing to the reduction of synthetic NH_3_ accumulation in the environment. But in developing these alternative solutions, it is necessary to design new catalysts which can follow alternative pathways that substitute the endergonic dissociative mechanism used in the Haber–Bosch process, and reduce the industrial energy spilt accounted for NH_3_ synthetic production.

## Nitrogenases

Nature, contrary to chemists, has found a way to use the abundant N_2_ gas effectively at room temperature and neutral pH by using natural catalysts, enzymes, called nitrogenases. The nitrogenase can channel electrons and energy from different sources in anaerobic and aerobic conditions to form bioavailable NH_3_ breaking the triple bond of the (almost inert) molecules of N_2_ gas. Three homologous nitrogenases have been reported, distinguished by their metal-centred catalytic cofactors: molybdenum (MoFe), iron (FeFe) and vanadium (VFe) (Hu and Ribbe, 2015). Although the three homologous enzymes have been associated with specific activities, our understanding of the nitrogenase metal cofactors and their role is still incomplete (Rutledge and Tezcan, 2020).

The more ancient, abundant, efficient, and studied nitrogenase is the molybdenum containing system (Curatti et al., 2006). This nitrogenase is composed of two proteins, an homodimeric iron (FeP) protein (∼66 kDa) and the α_2_β_2_ heterotetrameric molybdenum-iron (MoFe) protein (∼240 kDa, with two complex metalloclusters). The FeP protein contains an ATP-binding site within each subunit interface of the protein, and it oversees the shuttle of eight electrons towards the reduction of 1 mole of N_2_. Concomitantly, 1 mole of H_2_ is produced per mole of N_2_ fixed. The explanation for this H_2_ reduction and apparent waste of equivalent power remains elusive but considering that H_2_ reduction by nitrogenase occurs only in the presence of N_2_, it has been proposed that production of H_2_ activates the FeMo-protein. Together, the oxidation of the low-potential [4Fe-4S]^1+^ cluster requires activation, and this happens when the hydrolysis of ATP takes place (Barsukova-Stuckart et al., 2012). Commonly, the ATP requirement of nitrogenase is evaluated as 2 moles of ATP are hydrolyzed into ADP and inorganic phosphate (P_i_) per mole of electrons transferred, although more efficient ratios (down to 1 mole of ATP consumed per mole of electron) have been reported (Tan et al., 2016; Poudel et al., 2018). With this, the overall stoichiometry of natural N_2_ fixation remains as presented in Eq. 1.

$${\text{N}}_{2}{+8\text{ H}}^{+}+\text{8 e}¯+\text{nATP}↔{2\text{NH}}_{3}{+\text{H}}_{2}{+\text{nADP}+\text{nP}}_{\text{i}}$$ (1)

The detailed explanation for the necessary loss of a cell’s energy currency (ATP) associated with nitrogenase activity remains elusive (Rabo and Schoonover, 2001; Milton et al., 2017) but it is assumed to be essential to reduce the activation barriers associated to the catalysis of the intermediates that lead to the overall reaction (Van Der Ham et al., 2014) and to activate the transfer of electrons (Rutledge and Tezcan, 2020). Also, the electron transfer to the substrate in nitrogenase seems to follow the description drawn in 1978 by Thorneley and colleagues (Thorneley et al., 1978), but the delicate and precise donation of electrons, protons and energy is not fully deciphered yet. Meanwhile, this optimized coordinated mechanism plays a fundamental role in maintaining the high efficiency of the non-selective nitrogenase enzyme (Kang et al., 2021).

After the donation of electrons, the [4Fe-4S]^1+^ cluster must be reduced again. This can happen by subsequent reduction by flavodoxin in aerobic or facultative anaerobic organisms, or by ferredoxin (more sensitive to O_2_ presence) in anaerobic ones. Phylogenetic analyses suggested the use of flavodoxin as strategy for diversification of nitrogenases in aerobic environments (Boyd et al., 2015). The electrons that feed flavodoxin and/or ferredoxin come directly from pyruvate or H_2_ oxidation (mostly in anaerobic organisms) or NAD(P)H electron carriers (aerobic, facultative anaerobes, and anoxygenic phototrophs) (Poudel et al., 2018). Indeed, the reduction of flavodoxin or ferredoxin starts the cycle towards N_2_ fixation again.

Although some research efforts have been trying to take advantage of the high efficiency of nitrogenase using the two-protein mechanism to directly catalyse N_2_ fixation (Harris et al., 2018), the high efficiency of electrons donated per mole of N_2_ fixated by nitrogen-fixing bacteria (8 electrons per mole of NH_3_ produced), has not been achieved by any *in vitro* system using the MoFe protein, the nitrogenase enzyme or any inorganic catalysts (Table 1). Engineering of nitrogenase in eukaryotic cells is another promising avenue but still requires overcoming fundamental challenges (Yang et al., 2014; Vicente and Dean, 2017). Therefore, other efforts have been directed towards the generation of enzymatic fuel cells, which has been approached using methyl viologen as solely electron mediator between a cathodic surface and a nitrogenase (Milton et al., 2017). This is a rather difficult catalysis as it requires an ATP regenerating system to activate the FeP protein, and anaerobic conditions, with remarkably low efficiencies reported. To remove the necessity of an ATP regeneration, bioelectrocatalysis of N_2_ fixation has been explored using only the MoFe protein of the nitrogenase and cobaltocene as electron mediator (Milton et al., 2016). However, production of NH_3_ was only reported with the reduction of N_3_^−^ or NO_2_^−^.

**TABLE 1:** Efficiencies of selected novel catalysts for N_2_ fixation under ambient temperatures and pressures. RHE = Reversible Hydrogen Electrode / SCE = Standard Calomel Electrode.

Catalyst	Efficiency (mol NH_3_ e^−1^)	Ammonia rate (μgNH_3_ mg_cat._^−1^ h^−1^)	Electronic promotor	Ref
**Electrocatalysts**				
Ru SAs/N–C	0.0493	120.90	−0.20 V vs RHE	Geng et al. (2018)
Pd/C	0.0137	4.50	0.10 V vs RHE	Wang et al. (2018)
MoO_3_ nanosheets	0.0032	29.43	−0.50 V vs RHE	Han et al. (2018)
Bi_4_V_2_O_11_/CeO_2_	0.0169	23.21	−0.20 V vs RHE	Lv et al. (2018)
Mo_2_N nanorod	0.0075	78.40	−0.30 V vs RHE	Ren et al. (2018)
Au/TiO_2_	0.0135	21.40	−0.20 V vs RHE	Shi et al. (2017)
CN-C_500_	0.0280	2.90	−0.30 V vs RHE	Peng et al. (2020)
**Photocatalysts**				
Bi_2_MoO_6_	0.0012	22.14	Xe lamp (λ = 500 nm)	Hao et al. (2016)
**Synthetic electron donor**				
[Co(N_2_)(^tBu^PNP)	0.0442	47.22	KC_8_	Kuriyama et al. (2016)
**MOF-Polyoxometalate Catalysts PMo**_**12**_ **@MIL-100 (Fe) precursor**				
FeMo-based material	0.0912	105.30	−0.40 V vs RHE	Wang et al. (2020)
**Enzymatic Fuel Cells**				
MoFe / Cobaltocene	0.0583	12.72	−1.25 V vs SCE	Milton et al. (2016)
MoFe / Methyl viologen	0.0440	2.44	−0.85 V vs SCE	Milton et al. (2017)

Few electrochemical systems that produce convincing amounts of NH_3_ have been reported with the most successful so far being the molybdenum based ones (see, Table 1). However, the poor Faradaic efficiency of these systems due to their low selectivity competing with H_2_ production, makes them, in many cases more energy demanding than Haber–Bosh process (Van Der Ham et al., 2014). These inefficiencies can only be surpassed by the design of other catalysts able to follow a more feasible reaction pathway at room temperature. The reliability of experimental electrochemical nitrogen reduction reaction (ENRR) experiments was questioned in a recent publication by Choi et al. detailing the complexity that arises from the potential intrusion of airborne contaminants. The reduction of nitrogen oxides (NO, NO_2_, etc.,) are more thermodynamically favorable than direct ENRR (Choi et al., 2020). Failure to control this has led to contentious Faradaic efficiencies and ammonia yields.

The design of novel bio-inspired catalysts, containing multiple active sites, has the potential to bypass the obvious limitations associated with exploitation of the complex nitrogenase enzyme, although competitive CO_2_ and H_2_ selectivity must be overcome with concomitant effectiveness in N_2_ adsorption and mechanistic delivery of electrons and protons (Bagger et al., 2021). Other authors, have reported that the use of a bio-inspired catalysts operating via an associative mechanism, like the one described for nitrogenases, are able to fix N_2_, CO_2_ and CH_4_ simultaneously at room temperature (Revilla-López et al., 2020). This can open the avenue for the development of new industrial processes able to combine N_2_ fixation with carbon homologation.

## Molecular Metal Oxides or Polyoxometalates

Molecular metal oxides, or polyoxometalates (POMs) offer a route to design efficient ENRR using Earth abundant transition metals. POMs are primarily comprised of early-transition-metal (*d-block*) elements in their highest oxidation states. A great majority of these structures are anionic and consequently salts with charge balancing cations. In fact, POMs are an archetypal family of self-assembled molecular clusters that display a vast range of physical properties, structural features and sizes (Vilà-Nadal and Cronin, 2017). POMs are mainly formed by Mo^6+^ and W^6+^ combined with a main group oxyanion (phosphate, silicate, etc.,). Simply speaking, the synthesis of POM clusters in a “one-pot” solution involves dissolving the [MO_4_]^n−^ (M = W, Mo) salt in aqueous solution followed by acidification, addition of electrophiles, buffer, additional cations and in some cases a reducing agent (Proust et al., 2012). The solution can then be processed by normal, microwave or hydrothermal heating followed by controlled precipitation to yield the cluster in crystalline form so that the structure of the cluster can be elucidated by single crystal X-ray diffraction (Long et al., 2004).This route has been used in 99% of all cases in POM chemistry and is very convenient to yield complex structures from “one-pot” but suffers a great deal from dependence on initial reaction conditions, reproducibility, and the ability to systematically investigate parameter space to design new cluster architectures. In this respect, during the last decade the field of POMs has been transformed by trapping reactive building blocks and generating an accessible building block library as a function of pH, template, linker heteroatoms, and cation type (Miras et al., 2020). The key aspect here is that the heteroatom mediated assembly of the anionic metal-oxo units to building blocks which then link to clusters, can be used to form new types of materials with novel and unprecedented architectures (Zheng et al., 2018). In fact, POM structures and functionalities make them ideal candidates as model systems for metal-oxide-anchored single atom catalysts (POM-SAC) (Liu and Streb, 2021). POMs are polynuclear metal oxide anions that are molecular analogues of solid-state metal oxides. Diverse fields such as, water oxidation catalysts (Blasco-Ahicart et al., 2018), photocatalysis (Costa-Coquelard et al., 2010), molecular electronics (Busche et al., 2014), quantum computation (Gaita-Ariño et al., 2019), biology (Gumerova and Rompel, 2021) and medicinal science (Lu et al., 2021) have all been impacted by POM chemistry. Current findings demonstrate the feasibility of hydrogen-production using silicotungstic acid, H_6_[SiW_12_O_4_], by coupling low-pressure oxygen production via water oxidation linked to non-electrolyzer catalytic hydrogen production (Rausch et al., 2014). Given their structural diversity and versatility of POM cluster applications, they are ideal candidates to provide further insight into the heterogeneous Haber–Bosch catalyst or the low-energy nitrogenase enzymes that directly make ammonia.

## Discussion

Ammonia is a viable hydrogen energy vector, and its pre-existing industry, which produces, stores, and trades millions of tons of ammonia annually, means that the infrastructure necessary to jump-start the hydrogen economy already exists. The United Kingdom has developed detailed plans for the next decade to use “green” ammonia as an energy storage material for renewable electricity (The Royal Society, 2020).

The global cycling of nitrogen through the biosphere depends upon a heavy element: molybdenum and requires bacteria in the fixation of nitrogen (Hille, 2002). However, when extensively starved nitrogen-fixating bacteria *A. Viinelandi* were grown in a medium that lacked molybdate but that contained tungstate, *A. vinelandii* synthesized the regular storage protein but with tungstate. This is perhaps not surprising since tungsten, lies below molybdenum in the d-block, and is consequently expected to feature chemical properties related to those of molybdenum. Recent work indicated that molybdenum and tungsten-based enzymes are incredibly ancient and their enzymatic role and functionality has been preserved (Vitousek et al., 2002). It is thought that in the reducing environment of the primordial world tungsten-enzymes were favoured. In those days, oxygen atom transfer reactions were more challenging than in our oxic modern world, with its preference for molybdenum-enzymes (Schemberg et al., 2007). By deepening our understanding of the microbial populations that cycle nitrogen, we can find opportunities to deliver more efficient bioengineering solutions. To date, no one has systematically explored the new biotechnologies for nitrogen removal that can emerge from this new knowledge because a purely empirical exploration would require significant investigation. To achieve low-temperature, cost-effective and efficient electrochemical ammonia synthesis requires a multidisciplinary approach able to characterise natural biocatalysts (nitrogenases) that efficiently catalyse N_2_ reduction, as well as develop heterogeneous (molecular) catalytic systems informed by current computational theory developments in the area that can direct efficiently experimental investigation (Foster et al., 2018).

**FIGURE 1:**
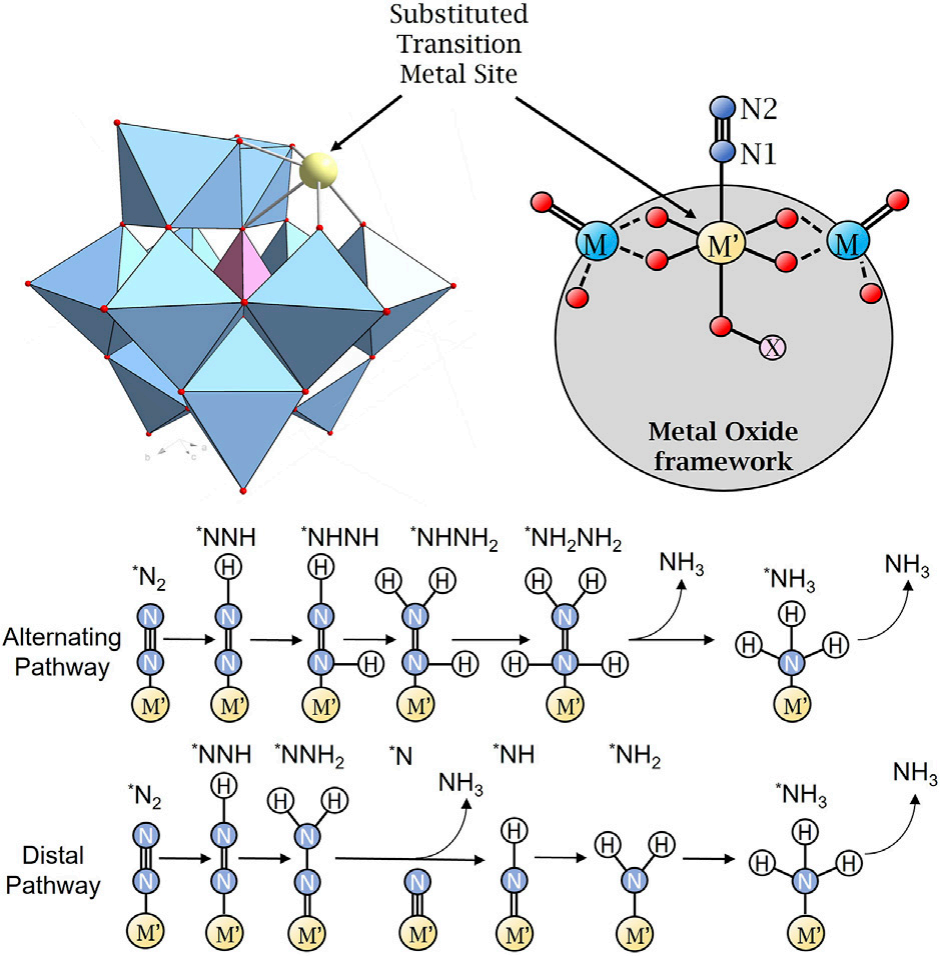
Polyhedral **(top-left)** representation of mono-substituted heteropolyanion. The nitrogen molecule adsorbs onto the transition metal site through the η^1^ (end-on) binding mode. The schematic depiction **(top-right)** shows the mono-substituted heteropolyanion in the nitrogen-bound state. Below this, a schematic depiction of the associative mechanisms for nitrogen reduction in which the N-N bond is cleaved simultaneously with the release of ammonia. The associative mechanism can proceed via two separate pathways—“alternating” and “distal” which invoke distinctly different intermediates. Colours corresponding to addenda metal = Cyan; substituted metal = yellow; heteroatom = Pink; O = red; N = dark blue; and H = white.

We will start by looking into transition metal substituted lacunary Keggin anions, as shown in Figure 1. Such structures are derivatives from the parent anion [XM_12_O_40_]^n−^, where X is the heteroatom (most commonly are P^5+^, Si^4+^, or B^3+^), and M = W, Mo, inspired by recent work in the area, (Lin et al., 2020) which investigated the Gibbs free energy change for the reductive adsorption of *N_2_ and *H on four Keggin-POM-supported Ru single atom electrocatalysts. The phosphorus-templated tungstate- and molybdate-Keggin clusters presented high nitrogen-binding selectivity, whereas the silicon-templated analogues prefer hydrogen binding. Our aim is to explore the functionalization of molecular dinitrogen and its catalytic conversion in POMs by combining our expertise in inorganic chemistry with exploring the catalytic conversion *d*-block metals. This will be our theoretical model structure, bearing in mind that the pH increases the Mo- and W-based Keggin ions gradually disintegrate (Kondinski and Parac-Vogt, 2018). Computational chemistry will help us to describe the intermediates of bioinspired reaction pathways. These results will complement in-depth metabolic analyses of highly efficient nitrogenases at ambient temperature and pressure. We will work closely with experimentalists in the area that will help us to translate our theoretical results into effective experimental N_2_ reduction catalysis.

## Data Availability

The original contributions presented in the study are included in the article/supplementary material, further inquiries can be directed to the corresponding authors.
